# Functional diversification of the nematode *mbd2/3 *gene between *Pristionchus pacificus *and *Caenorhabditis elegans*

**DOI:** 10.1186/1471-2156-8-57

**Published:** 2007-08-28

**Authors:** Arturo Gutierrez, Ralf J Sommer

**Affiliations:** 1 Max-Planck Institute for Developmental Biology, Department for Evolutionary Biology, Spemannstrasse 37, D-72076 Tübingen, Germany

## Abstract

**Background:**

Several members of the Methyl-Binding Domain protein family link DNA methylation with chromatin remodeling complexes in vertebrates. Amongst the four classes of MBD proteins, MBD2/3 is the most highly conserved and widespread in metazoans. We have previously reported that an mbd2/3 like gene (*mbd-2*) is encoded in the genomes of the nematodes *Pristionchus pacificus*, *Caenorhabditis elegans *and *Caenorhabditis briggsae*. RNAi knock-down of *mbd-2 *in the two *Caenorhabditis *species results in varying percentages of lethality.

**Results:**

Here, we report that a general feature of nematode MBD2/3 proteins seems to be the lack of a bona fide methyl-binding domain. We isolated a null allele of *mbd-2 *in *P. pacificus *and show that *Ppa-mbd-2 *mutants are viable, fertile and display a fully penetrant egg laying defect. This egg laying defect is partially rescued by treatment with acetylcholine or nicotine suggesting a specific function of this protein in vulval neurons. Using Yeast-two-hybrid screens, *Ppa*-MBD-2 was found to associate with microtubule interacting and vesicle transfer proteins.

**Conclusion:**

These results imply that MBD2/3 proteins in nematodes are more variable than their relatives in insects and vertebrates both in structure and function. Moreover, nematode MBD2/3 proteins assume functions independent of DNA methylation ranging from the indispensable to the non-essential.

## Background

DNA methylation is a common regulatory mechanism implicated in transcriptional silencing in vertebrates [1]. Methylated cytosines in DNA are recognized by a set of proteins carrying a methyl-binding domain (MBD) [2,3]. These MBD proteins interpret the methylation signal by recruiting components involved in transcriptional repression or heterochromatin formation [4,5].

In mammals, five members of the MBD family have been identified [6]. MeCP2 forms part of the Sin3/HDAC complex and, when mutated, causes a progressive neurological disorder known as the Rett syndrome (RTT) [7,8]. MBD1 couples DNA methylation with histone H3-K9 methyltrasferase activity mediated by SETDB1 [2,9]. MBD2 and MBD3 are core subunits of the Nucleosome Remodelling and histone Deacetylation (NuRD) complex [5,10-12]. Interestingly, mammalian MBD3, in contrast to the amphibian MBD3, is not able to bind methylated DNA [6,13]. Finally, MBD4 is a DNA N-glycosidase implicated in the repair of demethylated cytosines [14].

Outside the MBD, the five members of the family share no similarity in their primary sequence, with the exception of MBD2 and MBD3 [6,15-17]. Notably, invertebrates contain a smaller number of *mbd*-like genes. For example, in the nematode *Caenorhabditis elegans *and the fruit fly *Drosophila melanogaster*, only one gene with similarity to the mammalian *mbd2*/*mbd3 *genes has been identified. In *D. melanogaster *this gene is known as *mbd-2/3*, whereas in *C. elegans *this gene is named *mbd-2*, given the nomenclature rules of this organism [16,17]. In contrast, a recent report of the genome of the honeybee *Apis melifera *describes the presence of both *mbd-2/3 *and *mbd-1 *homologues [18].

In *D. melanogaster*, the *mbd2/3 *gene is alternatively spliced to produce two isoforms. The long isoform is expressed early in development and is able to bind specifically to CpT/A methylated DNA, in contrast to vertebrate MBD proteins which bind methylated CpG [19]. The short isoform, which is expressed in late embryogenesis, lacks part of the MBD and therefore is unable to bind methylated DNA [3,20]. A null allele of the gene *mbd2/3 *is viable and fertile but the stability of pericentric heterochromatin is considerably affected [19].

In contrast to *D. melanogaster*, no obvious DNA methylation system has been found in *C. elegans *and other tested nematodes [16]. The unique MBD2/3 protein, *Cel*-MBD-2, is characterized by the complete absence of a bona fide MBD. Knock down analysis using RNA interference (RNAi) showed variable phenotypes with different degrees of penetrance, such as paralysis and death due to rupture through the vulva [16]. In contrast, RNAi of the homologous gene in *C. briggsae *reveals an essential function since the worms arrest early in development [16].

Previously, we initiated the analysis of genes related to DNA methylation in another nematode, the diplogasterid *Pristionchus pacificus *[16]. *P. pacificus *has been used as a satellite organism to study the evolution of developmental processes and developmental mechanisms (for review see [21]). We showed that the *P. pacificus *genome encodes an *mbd-2 *gene. However, as in *C. elegans*, *Pp*a-MBD-2 also lacks a recognizable MBD implying a function independent of DNA methylation. Remarkably, the genome of *P. pacificus *also encodes a homolog of the *D. melanogaster *DNA methyltransferase 2, *dnmt-2*, [16], but this protein has been shown to function in the cytoplasm of eukaryotic cells as a tRNA methyltransferase [22,23].

Here, we report the presence of *mbd-2 *genes in nematodes from different evolutionary clades. All encoded proteins have in common the absence of a MBD. In order to extend the functional analysis of *mbd-2*-like genes, we isolated a deletion mutant in *P. pacificus*. *Ppa-mbd-2 (tu365) *mutant animals are viable and fertile, but display a completely penetrant defect in the egg laying system (egg laying defective, Egl). The Egl defect is partially rescued by treatment with acetylcholine or nicotine, suggesting that *Ppa*-MBD-2 functions in the neuronal innervations at the vulva muscles. A molecular approach using Yeast two hybrid (Y2H) analyses reveals interaction with diverse cellular proteins but fails to show interaction of any of the tested members of the NuRD complex. On the basis of these results, we propose that the absence of the MBD and the functional diversification of the MBD2/3 proteins represent a derived character in nematodes.

## Results and discussion

### Nematode genomes contain *mbd-2*-like genes

**Figure 1:**
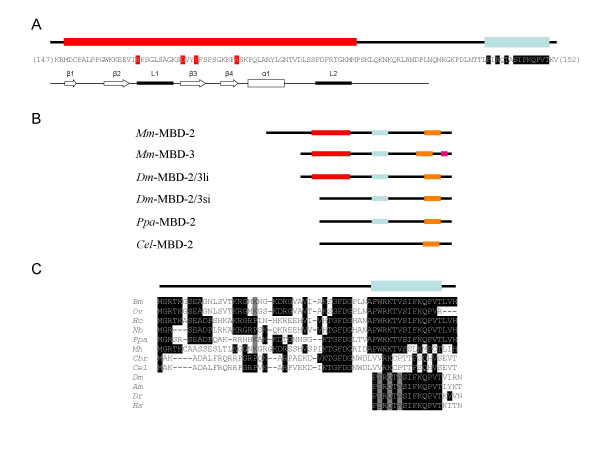
**Nematode MBD-2 proteins lack the methyl-binding-domain**. **A**. Amino-terminal sequence of the *H. sapiens *MBD-2 protein showing the methyl-binding domain (red box) and the conserved MBD2/3 motif (blue box). Critical residues interacting with methylated DNA are highlighted in red. The secondary structure of the methyl-binding-domain is shown below the sequence. Beta-strands 1–4 (β1–4), alpha helix 1 (α1), loops 1 and 2 (L1,2). **B**. Schematic alignment showing the MBD2 and MBD3 proteins from mouse (*Mus musculus*), the two isoforms of *Drosophila melanogaster *MBD2/3, *P. pacificus *and *C. elegans *MBD-2. Color codes refer to the methyl-binding-domain (red), the MBD2/3 SIFPQ conserved motif (blue), coiled-coil domain (orange) and an E-rich patch (pink) found only in MBD3. **C**. Amino acid alignment of the amino-terminal part of the MBD-2 proteins from *Brugia malayi *(Bm), *Haemochus contortus *(Hc), *Onchocerca volvulus *(Ov), *Meloidogyne hapla *(Mh) and *Nippostrongylus brasiliensis *(Nb), *Pristionchus pacificus *(Ppa), *Caenorhabditis elegans *(Cel) and *Caenorhabditis briggsae *(Cbr). The MBD2/3 conserved motif, SIFPQ, of *D. melanogaster, Apis mellifera, Danio rerio *and *Homo sapiens *MBD2/3 proteins is highly conserved in the nematode homologues except for *M. hapla *and the *Caenorhabditis *species.

The only member of the MBD family found in *C. elegans, C. briggsae and P. pacificus *belongs to the MBD2/3 group [16]. A key determinant of the MBD-2 proteins in these nematodes is the absence of a Methyl-Binding Domain (Fig. 1A,B; SFig. 1). To extend this observation to nematodes from different clades [24], we performed an extensive search of cDNAs in public genomic databases. Complete cDNA sequences were obtained for the filarial parasite *Brugia malayi *(clade III) and the rhabditid *Haemonchus contortus *(clade V). Partial 5' cDNA sequences were obtained for the spirurid *Onchocerca volvulus *(clade III), the tylenchid *Meloidogyne hapla *(clade IVa) and the rhabditid *Nippostrongylus brasiliensis *(clade V). Sequence comparison of the aminoterminal regions of the predicted MBD-2 proteins identified in different databases reveals a clear absence of an MBD (Fig. 1C). In addition, the highly conserved sequence motif SIFPQ, which is characteristic of the MBD2/3 group from vertebrates to nematodes, has changed substantially in the *Caenorhabditis *lineage and in *M. hapla *(Fig. 1C). We conclude that MBD-2 proteins are present in all studied nematodes, but that they lack a canonical methyl-binding domain.

### A *Ppa-mbd-2 *deletion mutant is viable and egg laying defective

The DNA methylation system encoded by members of the DNA methyl transferase family is essential in mammals, but is dispensable in lower vertebrates and invertebrates [22,25]. In particular, no *dnmt*-like genes are found in the genomes of *C. elegans *and *C. briggsae*. Because MBD proteins interpret methylation signals in insects and vertebrates, we hypothesized that the absence of an active DNMT protein and the unusual structure of the MBD protein in *Caenorhabditis *correlates with a functional diversification. Our observations, together with the fact that no DNA methylation system has been observed in any studied nematode, indicate that MBD-2 in nematodes evolved functions independent of this modification.

**Figure 2:**
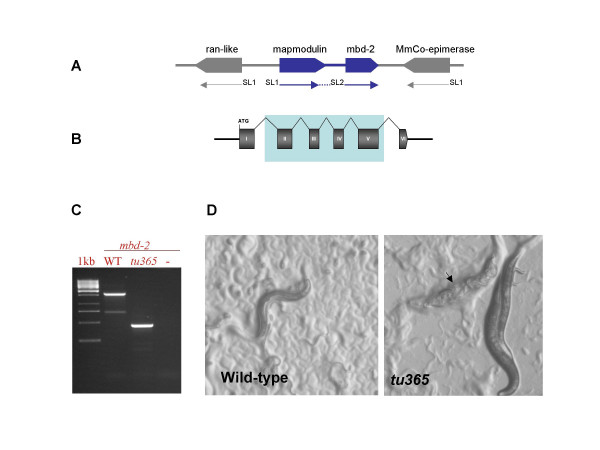
**A *P. pacificus mbd-2 *mutant is viable and Egg laying defective**. **A **The genomic locus of *Ppa-mbd-2*. The gene is part of an operon and is transspliced to SL2. The first gene of the operon, mapmodulin, is a microtubule binding protein. MnCo-epimerase: Methyl-malonyl CoA epimerase. **B ***Ppa-mbd-2 *gene structure. *Ppa-mbd-2 *is composed of six exons. Highlighted in blue are the exons removed in the deletion allele *tu365 *that cause the fusion of exon 1 and 6 and result in a frame shift. **C **Agarose gel showing the genomic amplification of the *Ppa-mbd-2 *gene with the primers AG10334–AG10335, indicating a deletion of approx. 1.7 kb in *tu365*. **D ***Ppa-mbd-2(tu365) *animals are egg-laying defective and form a "bag of worms" phenotype (arrow). See wild type animals for comparison.

To analyze the function of MBD-2 in nematodes other than *Caenorhabditis*, we used the satellite organism *P. pacificus*. This nematode is amenable to cellular and genetic studies and the genome is almost completely sequenced [21]. We applied a reverse genetics approach in *P. pacificus *to obtain a deletion mutant of the *Ppa-mbd-2 *gene using TMP/UV mutagenesis. We screened approximately 840,000 gametes and found one allele, *tu365*. This allele is likely to represent a null allele because four of the six exons are removed (ca. 1.7 Kb) and exon 1 is joined to exon 6 resulting in a frame-shift (Fig. 2A–C; SFig. 2).

The *Ppa-mbd-2(tu365) *mutant is recessive, homozygous viable and displays a fully penetrant Egl phenotype (Fig. 2D). Development proceeds normally until adulthood and gravid animals can lay an average of 40 eggs before eggs start to be retained by the mother. Eventually, egg retention and hatching inside the mother generate a "bag of worms" phenotype (Fig. 2D and Table 1). No paralytic-uncoordinated or rupture phenotype is seen in *Ppa-mbd-2(tu365) *mutants, as is the case in *Cel-mbd-2*(RNAi) treated animals.

**Table 1:** Early egg-laying behavior analysis of wild-type and *Ppa-mbd-2*(*tu365) *mutant animals

	Eggs-layed*
Wild-type	126 ± 10
*tu365*	41 ± 5

* Worms were synchronized by bleaching. Individual J2 were allowed to grow on normal NGM plates. The eggs laid by each worm were counted every 12 hours for a period of 48 hours. At each time point, every individual worm was transferred to a new plate. At 48 hours all the *tu365 *animals become egl. In both cases, 50 animals per strain were analyzed.

On the basis of these results, we argue that the function of MBD-2 in nematodes has changed from an essential to a dispensable gene. This argument is based on the observation that RNAi knock down of *mbd-2 *genes in the *Caenorhabditis *lineage produced lethality, whereas the *P. pacificus *null mutant is viable and fertile. It is important to note that *mbd-2 *genes are present in single copy in the genomes of *C. elegans*, *C. briggsae *and *P. pacificus*, as determined by Southern blot analysis (SFig. 3). Moreover, the location and structure of the *mbd-2 *gene differs in these nematodes. For example, in *C. elegans *and *C. briggsae*, the *mbd-2 *gene is located in an intron of the glycosylphosphatidylinositol anchor synthesis protein, C27A12.9, and is trans-spliced to the splice leader 1 (SL1). In *P. pacificus *in contrast, *mbd-2 *is the second gene of an operon and is trans-spliced to SL2, a key determinant of genes in operons (Fig. 2A) [26,27]. This difference suggests the potential for changes in the transcriptional regulation of the *mbd-2 *genes in these species and might represent a source for the acquisition of novel functions.

### *Ppa*-MBD-2 functions in the egg laying system

**Figure 3:**
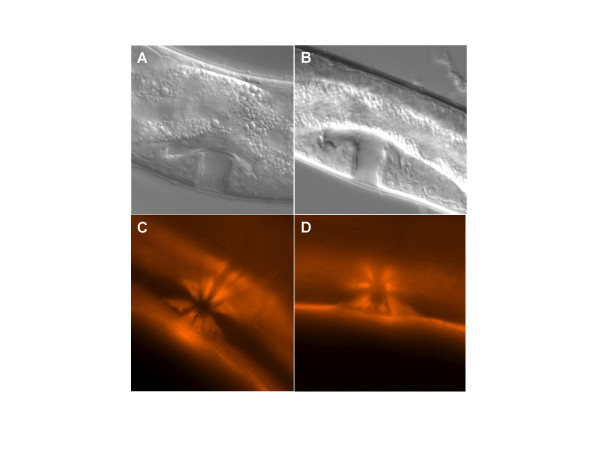
***Ppa-mbd-2 (tu365) *mutants have normal vulva and muscle development**. Adult wild-type (A, C) and *Ppa-mbd-2 *mutant animals (B, D) were analyzed by Nomarski microscopy (A, B) to determine vulva patterning and stained with phalloidin (C, D) to reveal sex muscles.

In *P. pacificus*, as in *C. elegans*, an Egl phenotype can be caused by many developmental or physiological defects. For example, misspecification of the vulva, the gonad, the sex musculature and several neurons innervating these muscles can result in an Egl phenotype. DIC-microscopy analysis of *Ppa-mbd-2(tu365) *mutant worms show that vulval cell fate specification is normal (Fig. 3A, B). Also, phalloidin staining, which stain actin fibers, revealed that the sex muscles are also present and correctly shaped (Fig. 3C, D). Therefore, the *Ppa-mbd-2 *Egl phenotype might result from defects in a neuronal function. As *tu365 *animals can initially lay eggs, we assumed that the neuronal system working on the egg-laying apparatus is formed properly and originally functional. Later on, neurodegeneration or failure in signal transmission might result in the Egl phenotype.

**Figure 4:**
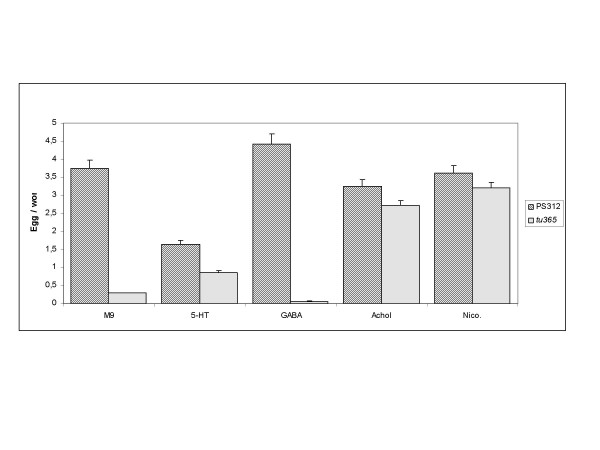
**Egg-laying assay of *P. pacificus *PS312 wild-type animals and *Ppa-mbd-2(tu365) *mutants**. Acetylcholine and nicotine partially rescue the Egl phenotype. Worms were incubated in a solution of M9 containing serotonin (5-HT), γ-aminobutiric acid (GABA), acetylcholine (AChol) or Nicotine (Nico.). Eggs were counted after two hours. At least 100 animals per treatment per strain were tested (± Standard deviation).

In *C. elegans*, the hermaphrodite specific neuron (HSN) and the ventral nerve cord neurons control the egg-laying program through the secretion of serotonin, acetylcholine and at least four neuropeptides [28]. Incubation of *C. elegans *with serotonin or nicotine stimulates egg laying [29,30]. In contrast, treatment with acetylcholine has opposing stimulatory and inhibitory roles in egg laying by acting through different types of receptors [31,32]. To test if the Egl phenotype of *Ppa-mbd-2(tu365) *can be rescued by neurotransmitters reported to work on the *C. elegans *egg-laying system, we incubated wild type and *Ppa-mbd-2(tu365) *mutant worms in M9 buffer containing different kinds of neurotransmitters or their agonists. It is important to note that wild type *P. pacificu*s PS312 animals, in contrast to *C. elegans*, retain no more than two to four eggs under normal cultural conditions. However, when worms were starved for about 12 hours, they retained on average six to eight eggs (environmental Egl). In our egg-laying assay, *P. pacificus *PS312 animals laid an average of 3.5 to 4.5 eggs per animal independent of the presence or absence of any of the tested neurotransmitters except for serotonin (5-HT). 5-HT has a negative effect on egg laying since wild-type induced environmental Egl animals resulted in a reduction of egg laying to an average of 1.5 eggs per animal (Fig. 4). Synchronized *Ppa-mbd-2(tu365) *mutant animals showed no or very low egg-laying in M9 and GABA. In contrast, treatment with acetylcholine and its agonist nicotine induced a potent (six-fold) increase in egg-laying in *Ppa-mbd-2(tu365) *(Fig 3). 5-HT treatment also increased egg-laying, but not as efficiently as acetylcholine and nicotine (Fig. 4). These results suggest that *Ppa-mbd-2 *may be implicated in cholinergic neurostimulation. More generally, these initial studies suggest a neuronal wiring that differs from the corresponding process in *C. elegans*, although we cannot completely rule out that the wiring is the same and that only the pharmacological features of the cells are different in both species. A more detailed study of the nervous system in *P. pacificus *will clarify these observations further. Interestingly, in a comparative approach using different nematode species, Loer and Rivard have shown recently that *P. pacificus *appear to have lost a serotonin-immunoreactive HSN [33]. Therefore, these results do not exclude the possibility that the neuronal function of MBD-2 is conserved in both species. One might speculate that MBD-2 could be required in a wider set of cell types in *C. elegans*.

### *Ppa*-MBD-2 interacts with diverse cellular proteins

Given that nematode MBD-2 proteins function independent of DNA methylation, they should have coevolved specific interaction partners. To test this hypothesis, we performed an unbiased Y2H screen using *Ppa*-MBD-2 as prey and a cDNA library of *P. pacificus *as bait (~1 × 10^7 ^clones). The protein does not show any auto-activation when present as both, bait and prey (Table 2). We found several different in frame interactors, such as first, a SPOP-like protein which is part of E3-ubiquitin ligase [34]; second, a myosin heavy chain homolog to the *C. elegans *T07C4.10 predicted protein; third, a Huntingtin interacting protein; fourth, a dynein ligh chain; fifth, a protein similar to *C. elegans *tag-260 and two proteins with no homology in the database (Table 2, SFig. 4). The fact that *mdb-2 *interacts with microtubule associated proteins such as dynein-light chain is interesting since the first gene of the operon in which *Ppa-mbd-2 *is placed encodes mapmodulin, a microtubule binding protein involved in vesicles transport from endosomes to trans-Golgi [35]. In this context it is important to note that genes arranged in operons have been suggested to display similar functions [26]. As some of these interactions may be physiological irrelevant, a final interpretation awaits a more detailed biochemical analysis.

**Table 2:** Yeast-2-Hybrid analysis of *Ppa*-MBD-2

Gene	-LTH+3AT^1^	LacZ
*mbd-2*	-	NA
dlc^2,3^	+	+
T07C4.10^2,3^	+	+
*bath-38*^2,3^	+	+
*tag-260*^2,3^	+	+
*hip-1*^2,3^	+	+
2 × No sim.^2,3^	+	+

^1 ^The assay was performed on plates depleted in leucine, triptophane and histidine, supplemented with 50 mM 3 AT. ^2 ^Results were confirmed by shifting prey and bait constructs. *mbd-2*, autoactivation control (pDEST-32-*Ppa-mbd-2*). ^3 ^Interactors found in the screen of the cDNA library. dlc, dineyn light chain. T07C4.10, myosin heavy chain homolog. Bath-38, BTB/MATH/POZ domain similar to Spop. Tag-260, proteins similar to *C. elegans *tag-260, annotated as a predicted E3-ubiquitin ligase. Hip-1, Huntingtin interacting protein, 2 × No sim, 2 clones with no homolog in the database. NA, not-applicable.

Interestingly, components of the NuRD complex were not found in the Y2H screening. However, sequencing of randomly chosen clones of the Y2H cDNA library revealed the presence of many in-frame components of the NuRD complex (data not shown). Therefore, we speculate that *Ppa*-MBD-2 is not involved in transcriptional repression or chromatin remodeling. Taken together, Y2H screens found *Ppa*-MBD-2 to associate with microtubule interacting and vesicle transfer proteins

## Conclusion

MBD proteins play a crucial role in DNA methylation and show an enormous multiplication and functional diversification in vertebrates. In insects, the number of genes encoding MBD proteins is smaller than in vertebrates. Our studies presented here indicate that in nematodes, only MBD2-like proteins are observed, which evolve rapidly at the sequence level. They show functions independent of DNA methylation, a phenomenon that is absent in *C. elegans*, *C. briggsae *and *P. pacificus*. With the limited functional data on MBD proteins from only three phyla (vertebrates, insects and nematodes) it remains currently unknown if the role of these proteins in DNA methylation represents the ancestral or derived function of the proteins. Importantly, MBD3 like proteins with a bona fide MBD are found in other lower metazoa such as in the platyhelminth *Schistosoma japonicum *[6]. Therefore, we speculate that the observed structure of the MBD in the MBD-2 proteins in the nematode lineage represents a secondary loss of a protein domain and similarly, that the functional diversification of nematode MBD2-like proteins represents a derived character. Thus, MBD-2 is an example of a protein that evolves rapidly and acquires new functions, including a role in epigenetics in higher metazoans.

## Methods

### Nematode strains

In the present study we used the strain PS312 of *Pristionchus pacificus *(Pasadena, CA) [36]. Worms were grown on an *E. coli *OP50 bacterial lawn at 20°C as previously described [36-38].

### Sequence analysis

Complete predicted MBD-2 protein sequences from the filarial parasite *Brugia malayi *and the rhabditid *Haemonchus contortus*, used for phylogenetic analysis, were derived from cDNA sequences obtained from the *B. malayi *database at the The Institute of Genome Research (TIGR) and Nematode.net, respectively. For alignments, partial cDNA sequences from *Onchocerca volvulus*, *Meloidogyne hapla *and *Nippostrongylus brasiliensis *were detected by TBlastn using the aminoacid sequence of *Ppa*-MBD-2 in the public available non-redundant database Nematode.net.

### TMP/UV mutagenesis and deletion library screening

TMP/UV mutagenesis and deletion screening were performed as described in Pires-daSilva, 2005 [39]. In short, synchronized *P. pacificus *larvae were washed off NGM plates with M9 and were incubated at 20°C for 15 min in a fresh solution of 30 μg/ml of Tri-methyl psoralen (TMP) (Sigma-Aldrich, USA). Next, worms were UV irradiated for 50 seconds under an intensity of 500 μW/cm^2^. Worms were recovered in OP50-NGM plates at a density of 200 worms per plate. After bleaching, 1400 plates with approximately 300 J2 stage larvae of the F1 generation were generated, resulting in a total of 840.000 gametes. After hatching of the F2 generation, 800 μl of distilled water was added and 150 μl were used for lysis. The remaining was left on the plates. Plates were stored at 12°C. For the deletion screen, 150 μl of worms suspension was combined with 1 volume worm lysis buffer to a final concentration of 10 mM Tris-HCl pH 8, 1 mM EDTA, 100 mM NaCl, 0.5% NP40, 0.5% Tween 20 and 100 μg/ml proteinase K. Lysis was done at 60°C for 4 hours and inactivated at 95°C for 15 minutes. DNA from14 plates were pooled in each well of a 96-well plate. Five microliters of DNA was used for PCR with the primers AG10332 CGCTTCTCACATGATCTTTGTC and AG10333 GGGTAGATTTGGAAGGTTGTTG. For the second PCR round, a 1/20 dilution of the first round reaction was amplified using the primers AG10334 AGAAAGAGTCCGAGGGAAACAC and AG10335 GGAGGGAAATGGTTATACTACAAGG. Positive candidates were retested at the single plate level. Sib selection was done using rounds of 100, 50 and 10 worms per plate/96-well plate. Finally, homozygous mutants were backcrossed six times to the PS312 strain.

### Behavioral assay

To characterize egg-laying response to different drugs, synchronized PS312 or mutant worms were grown in OP50 at 20°C until adult stage. Before starting the assay, PS312 worms were transferred to a NGM plate without food and incubated 12 hours at 20°C. Wild type and mutant Egl animals (that is, animals that retain in the uterus more eggs than normal) were placed individually in 100 μl of M9 buffer containing either 5 mg/ml 5-hydroxytriptamine, 3 mg/ml γ-aminobutiric acid (GABA), 5 mg/ml acetylcholine or 0.05% nicotine (Sigma-Aldrich, USA) [29-32,40]. All assays were performed with at least 100 animals/strain/drug at 20°C and eggs layed per worm were counted after 2 hours.

### Yeast-two-hybrid assay

The complete coding sequence corresponding to the gene *Ppa-mbd-2 *was amplified by reverse transcription PCR using the primers AG10576 ATGGCAAAAGCAGATGCTCTG and AG10577 CATCCAAAGTACTTCATAAC and cloned in pCR8-GW-Topo (Invitrogen, USA). Prey plasmid was done using LR-clonase into destination vector pDEST-32 BD clones (Invitrogen, USA,). Standard techniques were used to transform the plasmids into MaV203 yeast (Invitrogen, USA). Autoactivation test with this plasmid was negative. The prey pDEST-22 AD library of *P. pacificus *cDNA was prepared from poly(A) RNA using the Cloneminer kit following manufactures instructions (Invitrogen, USA,). Y2H screen was performed on SD plates lacking Leucine, Triptophan, Histidine plus 50 mM 3AminoThiazole. In total, 1 × 10^7 ^transformants were screened. Positive colonies were tested for LacZ staining, as reported [41]. In addition, positive clones were retransformed and retested.

## Abbreviations

MBD- 5'methyl cytosine binding domain.

DNMT- DNA methyl binding protein.

RNAi- RNA interference.

MeCP2- Methyl-cytosine protein 2.

RTT- Rett syndrome.

NuRD- Nucleosome remodeling and histone deacetylation complex.

SL- Splice leader- Egl: Egg laying defective.

HSN- Hermaphrodite specific neuron.

5-HT- 5-hydroxytriptamine (Serotonin).

GABA- Gamma-amoni butyric acid.

Y2H- Yeast two hybrid.

## Authors' contributions

AG designed and performed research, analyzed the data and wrote the paper. RJS designed research and wrote the paper. The authors read and approved the final manuscript.

## Supplementary Material

Additional file 1 **SFig. 1: *C. elegans *and *P. pacificus *MBD-2 proteins lack the methyl-binding-domain**. Protein alignment showing the MBD2 and MBD3 proteins from mouse (*Mus musculus*), the two isoforms of *Drosophila melanogaster *MBD2/3, *P. pacificus *and *C. elegans *MBD-2. Color codes refer to the methyl-binding-domain (red), the MBD2/3 SIFPQ conserved motif (blue), coiled-coil domain (yellow) and an E-rich patch (pink) found only in MBD3. **SFig. 2: Gene structure of the *P. pacificus mbd-2 (tu365) *mutant**. **A ***Ppa-mbd-2 *gene structure showing the exons deleted in the allele *tu365*. Blue arrows show the location of the primers used to obtain this mutant. **B **DNA sequence of the *mbd-2 *transcript in the mutant *tu365*. Note that the splicing of exon 1 and 6 creates an out-of-frame fusion ending after the residue 43. SL is the splice leader 2, typical of genes found inside operons. Primers AG10334 and AG10335 were used to do the RT-PCR shown in figure 2C. The red arrow below the sequence means the original starts and stop codons of the wild type gene. **SFig. 3: The genome of *Pristionchus pacificus *contains a single *mbd-2 *gene**. Genomic DNA was isolated from a mixed population of *P. pacificus *312 worms. DNA was digested with the enzymes *SalI *(S), *PstI *(P), *XhoI *(X), *ClaI *(C), *XbaI *(B) and *XhoI-PstI *(XP) for 4 hours at 37°C. The reactions were run in a 0.8% agarose gel and transferred to Nylon membranes. The hybridization was done overnight using a radiolabeled (^32^P) cDNA probe, representing the first 200 bp of the *mbd-2 *gene, in a solution containing 0,25 M Sodium phosphate, pH 7.2/7% SDS at 50°C. The membrane was washed two times at 50°C in a solution 20 mM sodium phosphate/5% SDS and exposed 12 hours to a Kodak BioMax XAR film. Increasing exposure times did not show additional bands above background. Molecular sizes are shown at the left of the southern. **SFig. 4: Yeast-two hybrid plaque assay**. Candidate colonies growing in a -LTH +3AT plates were transferred to a nitrocellulose filters. Filters were assayed for lacZ expression as in ref 41. The identity and number of clones belonging to a particular gene are described on the right panel. Click here for file

## References

[B1] Bird A (2002). DNA methylation patterns and epigenetic memory. Genes Dev.

[B2] Ohki I, Shimotake N, Fujita N, Jee J, Ikegami T, Nakao M, Shirakawa M (2001). Solution structure of the methyl-CpG binding domain of human MBD1 in complex with methylated DNA. Cell.

[B3] Ballestar E, Pile LA, Wassarman DA, Wolffe AP, Wade PA (2001). A Drosophila MBD family member is a transcriptional corepressor associated with specific genes. Eur J Biochem.

[B4] Berger J, Bird A (2005). Role of MBD2 in gene regulation and tumorigenesis. Biochem Soc Trans.

[B5] Wade PA, Gegonne A, Jones PL, Ballestar E, Aubry F, Wolffe AP (1999). Mi-2 complex couples DNA methylation to chromatin remodelling and histone deacetylation. Nat Genet.

[B6] Hendrich B, Tweedie S (2003). The methyl-CpG binding domain and the evolving role of DNA methylation in animals. Trends Genet.

[B7] Wan M, Lee SS, Zhang X, Houwink-Manville I, Song HR, Amir RE, Budden S, Naidu S, Pereira JL, Lo IF, Zoghbi HY, Schanen NC, Francke U (1999). Rett syndrome and beyond: recurrent spontaneous and familial MECP2 mutations at CpG hotspots. Am J Hum Genet.

[B8] Jones PL, Veenstra GJ, Wade PA, Vermaak D, Kass SU, Landsberger N, Strouboulis J, Wolffe AP (1998). Methylated DNA and MeCP2 recruit histone deacetylase to repress transcription. Nat Genet.

[B9] Sarraf SA, Stancheva I (2004). Methyl-CpG binding protein MBD1 couples histone H3 methylation at lysine 9 by SETDB1 to DNA replication and chromatin assembly. Mol Cell.

[B10] Feng Q, Zhang Y (2003). The NuRD complex: linking histone modification to nucleosome remodeling. Curr Top Microbiol Immunol.

[B11] Ng HH, Zhang Y, Hendrich B, Johnson CA, Turner BM, Erdjument-Bromage H, Tempst P, Reinberg D, Bird A (1999). MBD2 is a transcriptional repressor belonging to the MeCP1 histone deacetylase complex. Nat Genet.

[B12] Zhang Y, Ng HH, Erdjument-Bromage H, Tempst P, Bird A, Reinberg D (1999). Analysis of the NuRD subunits reveals a histone deacetylase core complex and a connection with DNA methylation. Genes Dev.

[B13] Fraga MF, Ballestar E, Montoya G, Taysavang P, Wade PA, Esteller M (2003). The affinity of different MBD proteins for a specific methylated locus depends on their intrinsic binding properties. Nucleic Acids Res.

[B14] Hendrich B, Hardeland U, Ng HH, Jiricny J, Bird A (1999). The thymine glycosylase MBD4 can bind to the product of deamination at methylated CpG sites. Nature.

[B15] Fatemi M, Wade PA (2006). MBD family proteins: reading the epigenetic code. J Cell Sci.

[B16] Gutierrez A, Sommer RJ (2004). Evolution of dnmt-2 and mbd-2-like genes in the free-living nematodes Pristionchus pacificus, Caenorhabditis elegans and Caenorhabditis briggsae. Nucleic Acids Res.

[B17] Tweedie S, Ng HH, Barlow AL, Turner BM, Hendrich B, Bird A (1999). Vestiges of a DNA methylation system in Drosophila melanogaster?. Nat Genet.

[B18] Wang Y, Jorda M, Jones PL, Maleszka R, Ling X, Robertson HM, Mizzen CA, Peinado MA, Robinson GE (2006). Functional CpG methylation system in a social insect. Science.

[B19] Marhold J, Kramer K, Kremmer E, Lyko F (2004). The Drosophila MBD2/3 protein mediates interactions between the MI-2 chromatin complex and CpT/A-methylated DNA. Development.

[B20] Marhold J, Zbylut M, Lankenau DH, Li M, Gerlich D, Ballestar E, Mechler BM, Lyko F (2002). Stage-specific chromosomal association of Drosophila dMBD2/3 during genome activation. Chromosoma.

[B21] Hong RL, Sommer RJ (2006). Pristionchus pacificus: a well-rounded nematode. Bioessays.

[B22] Goll MG, Kirpekar F, Maggert KA, Yoder JA, Hsieh CL, Zhang X, Golic KG, Jacobsen SE, Bestor TH (2006). Methylation of tRNAAsp by the DNA methyltransferase homolog Dnmt2. Science.

[B23] Rai K, Chidester S, Zavala CV, Manos EJ, James SR, Karpf AR, Jones DA, Cairns BR (2007). Dnmt2 functions in the cytoplasm to promote liver, brain, and retina development in zebrafish. Genes Dev.

[B24] Blaxter ML, De Ley P, Garey JR, Liu LX, Scheldeman P, Vierstraete A, Vanfleteren JR, Mackey LY, Dorris M, Frisse LM, Vida JT, Thomas WK (1998). A molecular evolutionary framework for the phylum Nematoda. Nature.

[B25] Lin MJ, Tang LY, Reddy MN, Shen CK (2005). DNA methyltransferase gene dDnmt2 and longevity of Drosophila. J Biol Chem.

[B26] Blumenthal T, Evans D, Link CD, Guffanti A, Lawson D, Thierry-Mieg J, Thierry-Mieg D, Chiu WL, Duke K, Kiraly M, Kim SK (2002). A global analysis of Caenorhabditis elegans operons. Nature.

[B27] Lee KZ, Sommer RJ (2003). Operon structure and trans-splicing in the nematode Pristionchus pacificus. Mol Biol Evol.

[B28] Schafer WF (2006). Genetics of egg-laying in worms. Annu Rev Genet.

[B29] Bastiani CA, Gharib S, Simon MI, Sternberg PW (2003). Caenorhabditis elegans Galphaq regulates egg-laying behavior via a PLCbeta-independent and serotonin-dependent signaling pathway and likely functions both in the nervous system and in muscle. Genetics.

[B30] Kim J, Poole DS, Waggoner LE, Kempf A, Ramirez DS, Treschow PA, Schafer WR (2001). Genes affecting the activity of nicotinic receptors involved in Caenorhabditis elegans egg-laying behavior. Genetics.

[B31] Bany IA, Dong MQ, Koelle MR (2003). Genetic and cellular basis for acetylcholine inhibition of Caenorhabditis elegans egg-laying behavior. J Neurosci.

[B32] Trent C, Tsung N, Horvitz HR (1983). Egg-laying defective mutants of the nematode Caenorhabditis elegans. Genetics.

[B33] Loer CM, Rivard L (2007). Evolution of neuronal patterning in free-living rhabditid nematodes I: Sex-specific serotonin-containing neurons. J Comp Neurol.

[B34] Furukawa M, He YJ, Borchers C, Xiong Y (2003). Targeting of protein ubiquitination by BTB-Cullin 3-Roc1 ubiquitin ligases. Nat Cell Biol.

[B35] Itin C, Ulitzur N, Muhlbauer B, Pfeffer SR (1999). Mapmodulin, cytoplasmic dynein, and microtubules enhance the transport of mannose 6-phosphate receptors from endosomes to the trans-golgi network. Mol Biol Cell.

[B36] Sommer RJ, Sternberg PW (1996). Evolution of nematode vulval fate patterning. Dev Biol.

[B37] Brenner S (1974). The genetics of Caenorhabditis elegans. Genetics.

[B38] Sommer RJ (1996). Morphological, genetic and molecular description of Pristionchus pacificus sp. n. (Nematoda : Neodiplogastridae. Fundamental and applied Nematology.

[B39] Pires-daSilva A, WormBook  (2006). Pristionchus pacificus genetic protocols.

[B40] Brundage L, Avery L, Katz A, Kim UJ, Mendel JE, Sternberg PW, Simon MI (1996). Mutations in a C. elegans Gqalpha gene disrupt movement, egg laying, and viability. Neuron.

[B41] Sambrook J, Russell DW (2001). Molecular cloning: A laboratory manual.

