# Supplementary material for: Functional diversification of the nematode mbd2/3 gene between Pristionchus pacificus and Caenorhabditis elegans
Source: BMC Genet. 2007 Aug 28;8:57. doi: 10.1186/1471-2156-8-57 (PMC2000911; doi:10.1186/1471-2156-8-57)
Supplement: Additional file 1 — SFig. 1: C. elegans and P. pacificus MBD-2 proteins lack the methyl-binding-domain. Protein alignment showing the MBD2 and MBD3 proteins from mouse (Mus musculus), the two isoforms of Drosophila melanogaster MBD2/3, P. pacificus and C. elegans MBD-2. Color codes refer to the methyl-binding-domain (red), the MBD2/3 SIFPQ conserved motif (blue), coiled-coil domain (yellow) and an E-rich patch (pink) found only in MBD3. SFig. 2: Gene structure of the P. pacificus mbd-2 (tu365) mutant. A Ppa-mbd-2 gene structure showing the exons deleted in the allele tu365. Blue arrows show the location of the primers used to obtain this mutant. B DNA sequence of the mbd-2 transcript in the mutant tu365. Note that the splicing of exon 1 and 6 creates an out-of-frame fusion ending after the residue 43. SL is the splice leader 2, typical of genes found inside operons. Primers AG10334 and AG10335 were used to do the RT-PCR shown in figure 2C. The red arrow below the sequence means the original starts and stop codons of the wild type gene. SFig. 3: The genome of Pristionchus pacificus contains a single mbd-2 gene. Genomic DNA was isolated from a mixed population of P. pacificus 312 worms. DNA was digested with the enzymes SalI (S), PstI (P), XhoI (X), ClaI (C), XbaI (B) and XhoI-PstI (XP) for 4 hours at 37°C. The reactions were run in a 0.8% agarose gel and transferred to Nylon membranes. The hybridization was done overnight using a radiolabeled (32P) cDNA probe, representing the first 200 bp of the mbd-2 gene, in a solution containing 0,25 M Sodium phosphate, pH 7.2/7% SDS at 50°C. The membrane was washed two times at 50°C in a solution 20 mM sodium phosphate/5% SDS and exposed 12 hours to a Kodak BioMax XAR film. Increasing exposure times did not show additional bands above background. Molecular sizes are shown at the left of the southern. SFig. 4: Yeast-two hybrid plaque assay. Candidate colonies growing in a -LTH +3AT plates were transferred to a nitrocellulose filters. Filters were as [file 1471-2156-8-57-S1.ppt]

## Slide 1
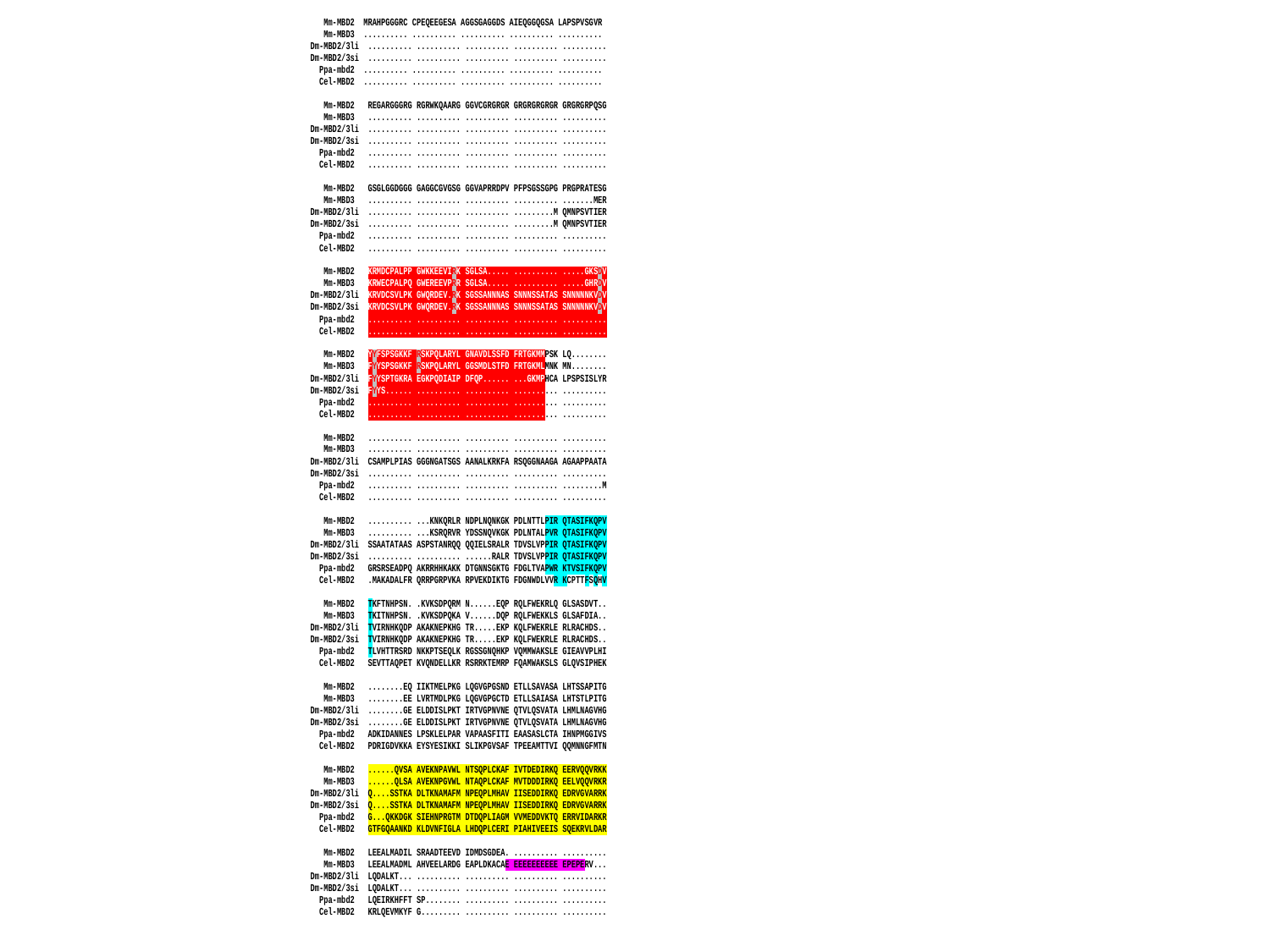

## Slide 2
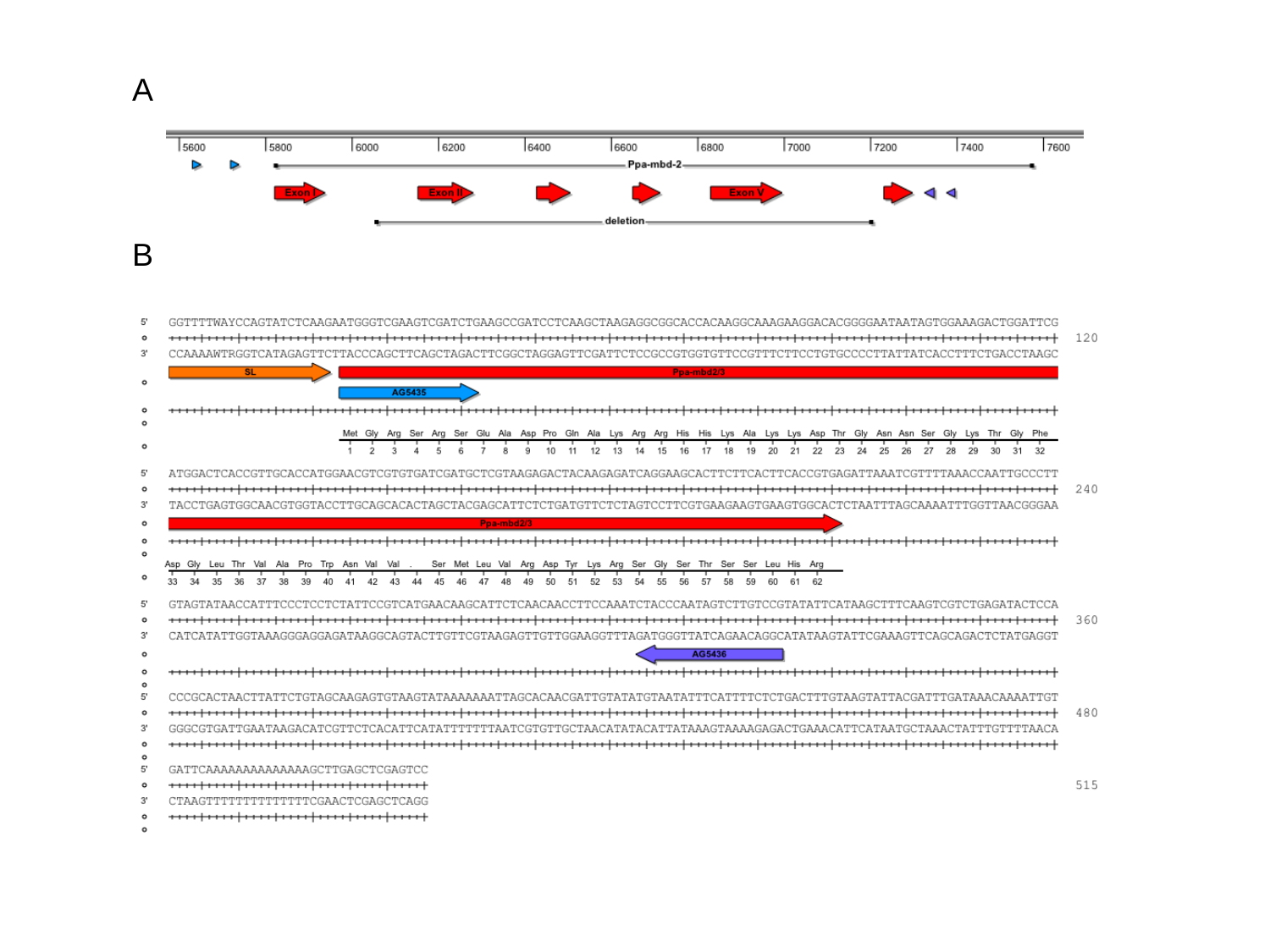

A
B

## Slide 3
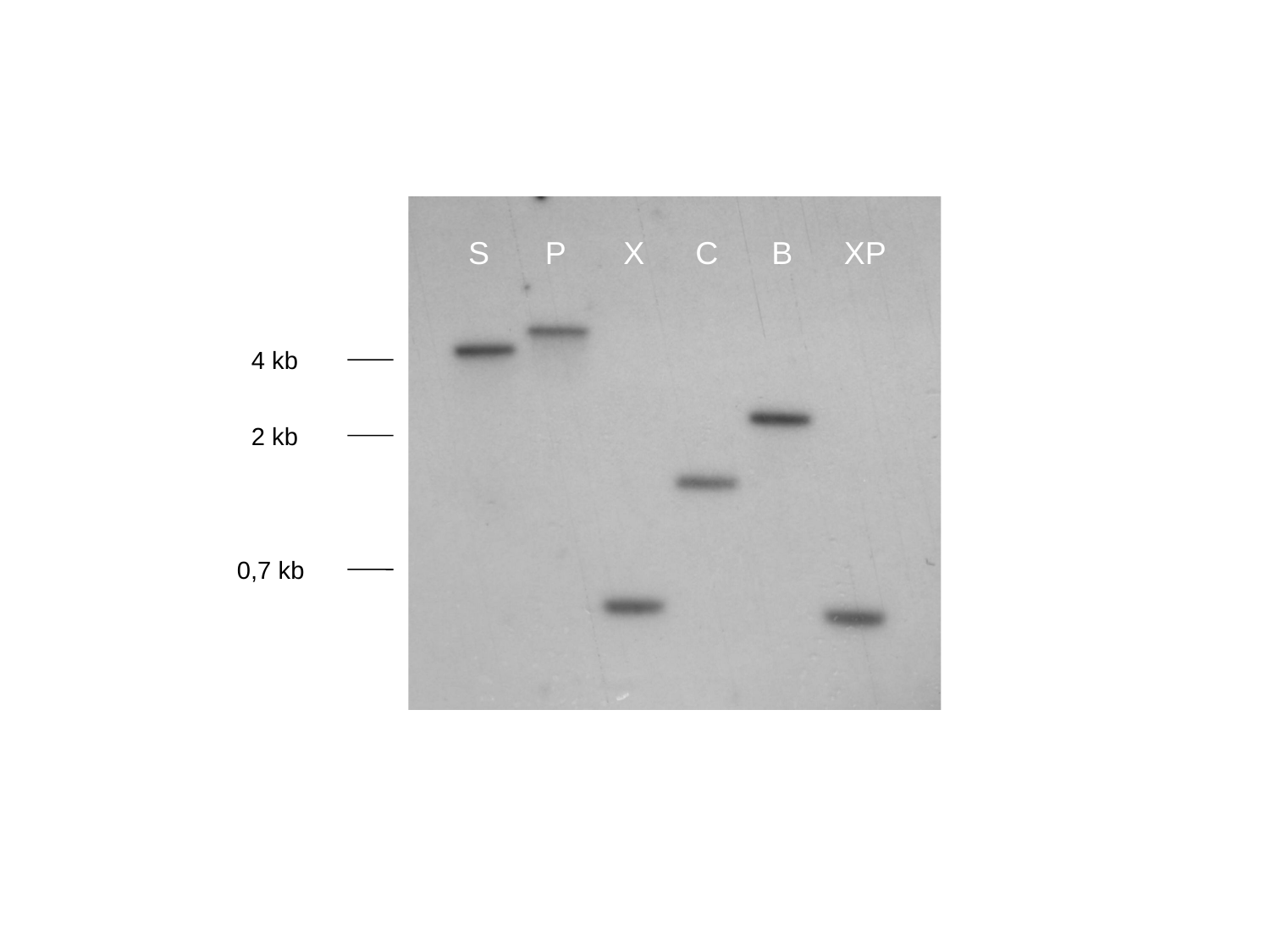

S
P
X
C
B
XP
4 kb
2 kb
0,7 kb

## Slide 4
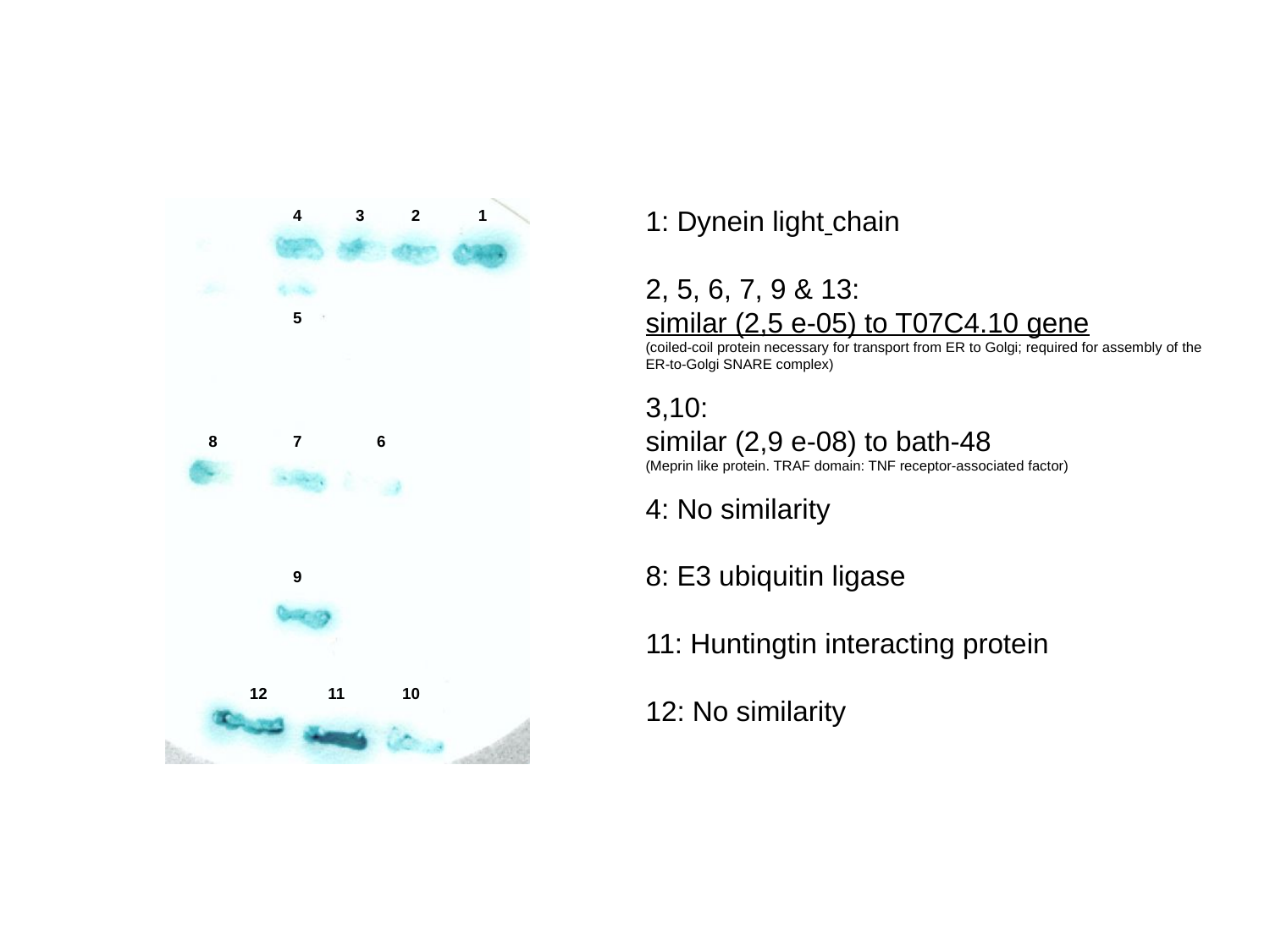

1: Dynein light chain
2, 5, 6, 7, 9 & 13:
similar (2,5 e-05) to T07C4.10 gene
(coiled-coil protein necessary for transport from ER to Golgi; required for assembly of the
ER-to-Golgi SNARE complex)
3,10:
similar (2,9 e-08) to bath-48
(Meprin like protein. TRAF domain: TNF receptor-associated factor)
4: No similarity
8: E3 ubiquitin ligase
11: Huntingtin interacting protein
12: No similarity
4
3
2
1
5
8
7
6
9
12
11
10
